# Extensive and differential platinum chemotherapy mutagenesis in livers of children

**DOI:** 10.1126/science.ady0339

**Published:** 2026-09-10

**Authors:** Anna Wenger, Jean-Baptiste Vannier, Henry Lee-Six, Robin Loesch, Giulia Emanuelli, Manas Dave, Mehdi Layeghifard, Andrew R.J. Lawson, Federico Abascal, Pantelis A. Nicola, Taryn D. Treger, Toochi Ogbonnah, Conor Parks, Thomas R.W. Oliver, Jonathan Kennedy, Angus Hodder, Nathaniel D. Anderson, Felipe Luz Torres Silva, Mi K. Trinh, Thomas Dowe, Marwo Habarwaa, James J. Sun, Sergio Assia-Zamora, Miriam Cortes-Cerisuelo, Wayel Jassem, Charlotte Town, Anil Dhawan, Vandana Jain, Karin Straathof, Maesha Deheragoda, Iñigo Martincorena, Liina Palm, J. Ciaran Hutchinson, Tim H.H. Coorens, Claire Trayers, Mariia Yuneva, Nigel Heaton, Adam Shlien, Yoh Zen, Foad J. Rouhani, Sam Behjati

**Affiliations:** 1 Wellcome Sanger Institute; Hinxton, CB10 1SA, UK; 2 Department of Laboratory Medicine, Sahlgrenska Center for Cancer Research, Institute of Biomedicine, Sahlgrenska Academy, University of Gothenburg; Gothenburg, 413 90, Sweden; 3 Tissue Regeneration and Clonal Evolution Laboratory, The Francis Crick Institute; London, NW1 1AT, UK; 4 Cambridge University Hospitals NHS Foundation Trust; Cambridge, CB2 0QQ, UK; 5 Oncogenes and Tumour Metabolism Laboratory, The Francis Crick Institute; London, NW1 1AT, UK; 6 Program in Genetics and Genome Biology, The Hospital for Sick Children; Toronto, ON, M5G 1X8, Canada; 7 Department of Paediatrics, University of Cambridge; Cambridge, CB2 0QQ, UK; 8 Great Ormond Street Hospital for Children; London, WC1N 3JH, UK; 9 University College London (UCL) Cancer Institute, University College London; London, WC1E 6BT, UK; 10 Department of Genetics, Institute of Biological Sciences, Federal University of Amazonas (UFAM); Manaus, Amazonas, 69080900, Brazil; 11 Liver Transplant Unit, King’s College Hospital NHS Foundation Trust; London, SE5 9RS, UK; 12 King’s Health Partners Centre for Translational Medicine; London, SE1 9RT, UK; 13 Roger Williams Institute of Liver Studies, School of Immunology & Microbial Sciences, Faculty of Life Sciences and Medicine, King’s College London, Foundation for Liver Research and King’s College Hospital; London, SE5 9NT, UK; 14 Pediatric Liver, GI and Nutrition Center and Mowat Labs, King’s College Hospital; London, SE5 9RS, UK; 15 European Bioinformatics Institute, European Molecular Biology Laboratory (EMBL-EBI); Cambridge, CB10 1SD, UK; 16 Broad Institute of MIT and Harvard; Cambridge, 02142 MA, USA; 17 Laboratory Medicine and Pathobiology, University of Toronto; Toronto, ON, M5S 1A8, Canada

## Abstract

Childhood cancer survivors often experience late adverse effects which may be linked to chemotherapy mutagenesis. We studied chemotherapy mutagenesis in pediatric normal tissues using duplex sequencing (NanoSeq) to enable the detection of mutations from single DNA molecules. We found that platinum chemotherapeutics increased mutation burdens of pediatric normal tissues to levels seen in adults. In the liver, platinum agents imparted a tissue-specific mutational signature that was absent from other tissues. Gene focused duplex sequencing revealed that chemotherapy mutagenesis generates a great diversity of non-synonymous variants, some of which may have functional potential such as leukemogenic variants in blood. Our findings demonstrate extensive chemotherapy mutagenesis in normal tissues of children, which may provide a plausible link between chemotherapy exposure and adverse effects in later life.

## Introduction

**Fig. 1:**
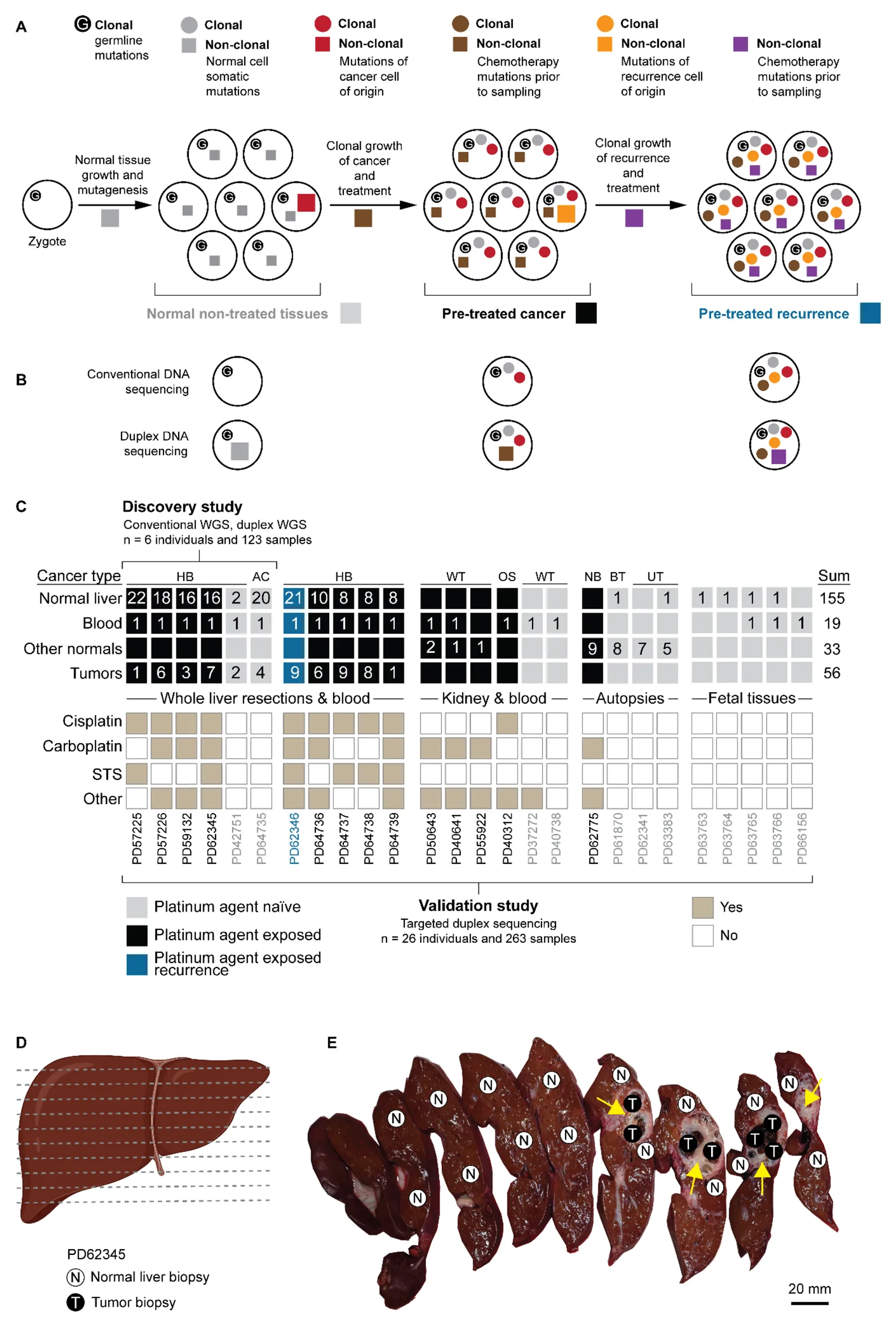
Conventional vs duplex sequencing, and study overview. **(A)** Schematic showing how mutations are accrued from the fertilized egg to a tumor recurrence. The fertilized egg harbors germline mutations which pervade all cells of the body. Normal cells acquire somatic mutations as they divide due to imperfect DNA replication (gray squares). Cancer-causing mutations (red square) may transform one of these cells, which then becomes the cancer cell of origin that clonally expands into a tumor, and the somatically acquired mutations thus become clonal (gray circle). All tumor cells carry the mutations of the cancer cell of origin (red circle) and acquire additional cell-specific (non-clonal) mutations as they diversify and from mutagenic chemotherapy (brown square). One of the cells will be the recurrence cell of origin and grow clonally into the recurrent tumor (blue in schematic and subpanel C) and all cells will therefore carry the mutations from that cell (orange circle), and will gain private non-clonal mutations induced by the additional treatment (purple square). **(B)** The mutations that are detected by conventional DNA sequencing (top) and duplex DNA sequencing (bottom) in normal tissues, pre-treated cancer, and pre-treated recurrence respectively. Note that non-clonal mutations (present in individual cells) can be detected by duplex sequencing whereas mutations need to become clonal to be detected by conventional DNA sequencing (for example, the non-clonal somatic mutations, gray square, present in the cancer cell of origin will become clonal in the pre-treated cancer, gray circle, and thus detectable by conventional DNA sequencing). **(C)** Overview of samples and children in the study detailing the number and type of samples, sequencing assays, and the treatment for each child. The blood sample for PD57226 was not taken at the same time point as the liver was sampled. HB: hepatoblastoma, AC: metastatic adrenocortical carcinoma in the liver, WT: Wilms tumor, OS: osteosarcoma, NB: neuroblastoma, BT: benign fetal lung tumor, UT: undifferentiated tumor, STS: sodium thiosulfate, Other: other treatment than cisplatin/carboplatin/STS. **(D)** Liver schematic of sampling of PD62345. Created with Biorender. **(E)** Picture shows the fresh, unfixed liver of PD62345 after resection and prior to sampling. The sampling strategy is shown with each circle representing the site of tissue sampling. Normal liver (N) and tumor (T) tissue. Yellow arrows delineate the tumor.

Some chemotherapeutics confer cytotoxic effects through damage of DNA (1). When this damage is not lethal to cells, agent-specific mutational imprints (signatures) (2) may emerge in surviving cells if DNA repair is imperfect. Past investigations have documented a variety of chemotherapy mutational signatures in both cancer (typically in the form of recurrences or metastases) and clonal expansions of normal cells (2–7). Chemotherapy mutagenesis in normal tissues has mainly been studied in blood through high depth sequencing of hematopoietic cancer genes. This approach has identified clonal expansions (clonal hematopoiesis) (8) which, in some adult individuals with prior cancer treatment, were characterized by mutational signatures attributed to specific chemotherapy agents (9, 10). Chemotherapy mutagenesis has been further substantiated by DNA sequencing of individual blood cells, expanded through single cell cloning for high fidelity DNA amplification, demonstrating that it pervades blood cells even in the absence of clonal expansions (11, 12). Similarly, investigations of genomes of relapsed cancers, which are reporters of past treatment and exposures (Fig. 1A), have corroborated mutational signatures attributable to chemotherapy, in particular to platinum-based agents (3, 4). Recent studies of tissues other than blood indicate that chemotherapy mutagenesis affects human tissues more broadly (6, 13–17). It is unknown whether the same exposure generates different mutations across tissues within an individual. This is a particularly pertinent question in children, in whom the consequences of potentially harmful mutagenesis, such as second cancers, may unfold over the span of a lifetime (18–20).

The platinum-based agents cisplatin and carboplatin have emerged as principal exemplars of mutation-inducing chemotherapeutics (4, 21). They are the backbone of many treatment protocols, particularly for solid childhood tumors. Unlike other mutagenic agents (such as the purine analogue, thiopurine), mutagenesis by cisplatin and carboplatin is independent of the cell cycle (22–24) and seems to be pervasive in adults in both normal and cancerous tissues following treatment (2, 3, 6). The extent and nature of platinum mutagenesis in children remain largely unknown. The largest study to date, examining 2,860 childhood cancer survivors, found no platinum signals in blood (25), whilst another study of cancer survivors, which included some children, found only subtle effects (26). By contrast, there have been occasional reports of platinum agent mutagenesis in blood (24, 27) and other normal tissues (13) of exposed children. Furthermore, studies of recurrent or second cancers in children have demonstrated mutagenesis by cisplatin and carboplatin (13, 27–30). The discrepancy can probably be attributed to technological limitations of conventional DNA sequencing, which depends on the presence of clonal outgrowth for mutation detection (Fig. 1A-B; Supplementary Text) (31). This shortcoming can be overcome by methods that enable precise mutation calling from single DNA molecules (32), independent of clonal expansions, through barcoding of both strands of DNA (duplex sequencing) to differentiate true mutations (present on both strands) from artefacts (present on one strand) (Fig. 1A-B). Using this approach, we examined platinum mutagenesis in children, focusing on liver and blood, supplemented with a variety of other normal tissues obtained at post mortem and surgical resections.

## Results

### Overview of experiment

Initially, our investigation focused on children with hepatoblastoma who underwent whole liver replacements as part of their treatment, which enabled us to study multiple areas of normal liver, tumor, and matched blood from the same child (Fig. 1C-E). Prior to surgery, children with hepatoblastoma are typically treated with cisplatin, either alone or in combination with carboplatin and other agents. In addition, we studied children with other cancer types (Wilms tumor or osteosarcoma) who had received platinum-based chemotherapy prior to resection. For comparison, we included children who had received non-platinum agents as well as treatment-naïve children, whose tissues we sampled from surgical specimens or at post-mortem, and human fetal livers as additional treatment-naïve controls (Fig. 1C). As fetal livers contain a large amount of hematopoietic islands, we were able to examine both liver and hematopoietic cells by separating the respective cell populations from each other. Our principal tool for mutation detection was an established implementation of duplex sequencing (NanoSeq) (32), to examine genome-wide mutations (whole genome NanoSeq), as well as mutations within specific genes (targeted NanoSeq (33)). The latter enabled us to determine potential functional consequences of variants. We called mutations using established variant calling pipelines (32, 34) (detailed in Methods). An overview of the tissues we studied and our experimental strategy is shown in Fig. 1C and is detailed in Table S1.

### Increased mutation burden in platinum-exposed liver tissues

**Fig. 2:**
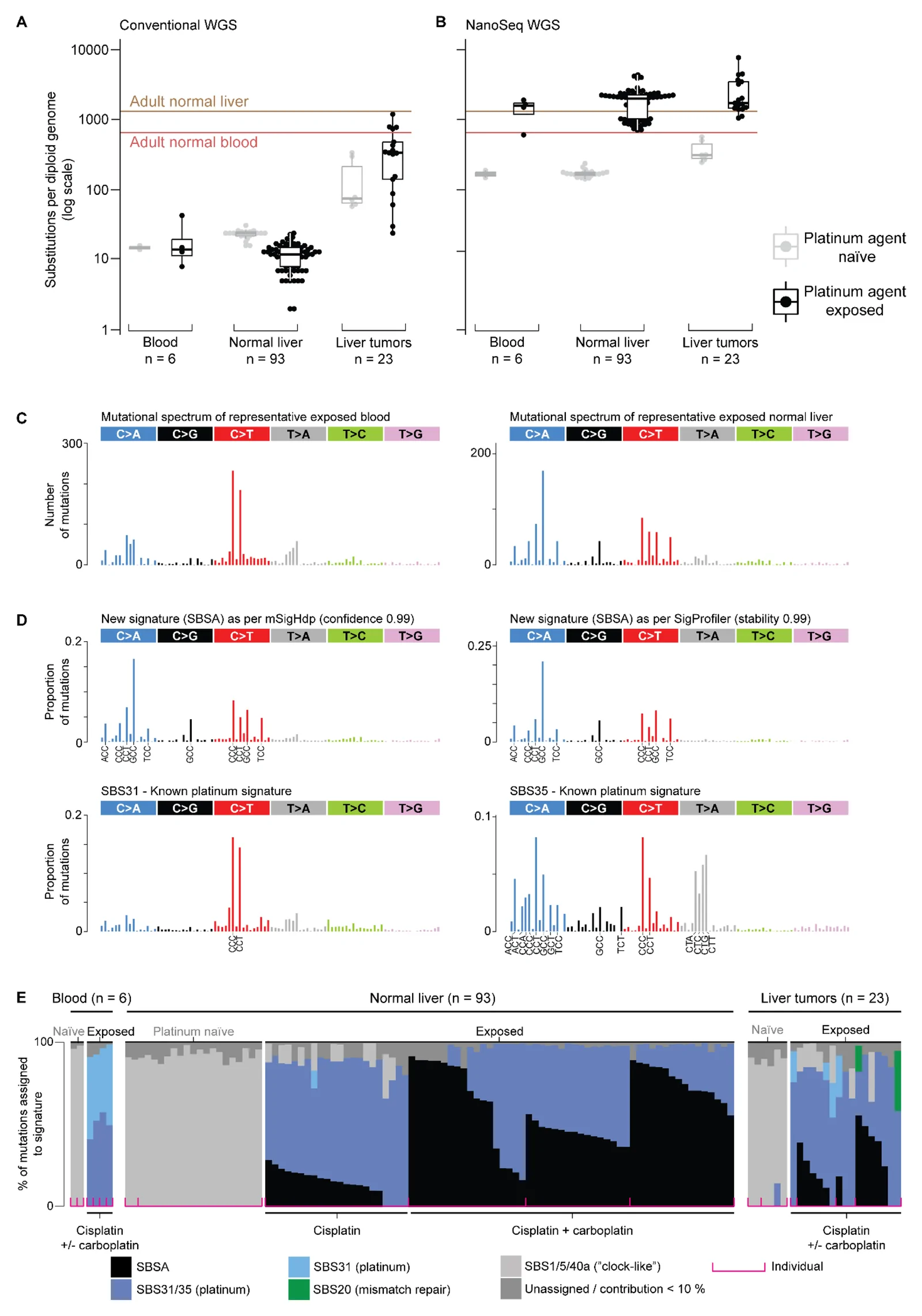
Whole genome mutation burden and signature analysis in discovery cohort. **(A)** Mutation burden by conventional whole genome sequencing (WGS). X-axis: different tissues. Y-axis: number of single base substitutions per diploid genome (log scale). Red and brown horizontal lines represent mutation burdens observed in normal adult blood (36) and liver (35). **(B)** Mutation burden by whole genome NanoSeq. Axes and horizontal lines as per (A). **(C)** Plots showing the normalized trinucleotide context (the reference base before and after a variant) of each single base substitution (X-axis) and the frequency of each trinucleotide for platinum-exposed blood (left) and platinum-exposed normal liver sample (right), which differ visually and according to the de novo signature extractions (see (D)-(E)), where SBSA was absent from blood while up to 92 % of the mutations per normal liver sample were assigned to SBSA. **(D)** Trinucleotide context for: Upper plots – a new signature, SBSA, as extracted from whole genome NanoSeq data by mSigHdp (left) and SigProfilerExtractor (right). Lower plots - Known signatures of platinum agents in COSMIC database (SBS31 and SBS35). **(E)** Contribution of signatures to each sample (sequenced with whole genome NanoSeq). X-axis: Each stacked bar plot represents a tissue sample as per labelling. Y-axis: % of mutations assigned to signature. Pink square brackets delineate individuals. Colors represent mutational signatures as per legend. The mSigHdp extraction is shown here. Similar results were obtained by SigProfilerExtractor (Fig. S4B). Note that SBSA was only evident in platinum-exposed livers whereas SBS31 and SBS35 were pervasive across all platinum-exposed tissues. See representative spectra in (C).

In a first experiment we studied normal livers (n = 93 samples), blood (n = 6 samples), and corresponding tumors (19 hepatoblastoma samples and 4 liver metastasis samples of an adrenocortical carcinoma) of 4 pre-treated and 2 chemotherapy-naïve children (discovery cohort; Fig. 1C). Within the normal liver we systematically sampled all anatomical regions (Fig. 1D-E) and interrogated DNA by conventional whole genome sequencing (WGS) and by whole genome NanoSeq. Conventional WGS of normal liver and blood yielded, as expected, occasional single base substitutions consistent with mosaic variants, with an average of 14 substitutions (range 2 - 31) per sample (Fig. 2A; Table S2). Conventional WGS of tumors showed typical cancer genomes with hundreds of substitutions as well as other expected somatic changes (insertions/deletions (indels), common copy-number changes for hepatoblastoma such as chromosome 11p LOH) including typical cancer-causing (driver) mutations (Fig. S1; Table S3). By contrast, whole genome NanoSeq revealed an extraordinarily high substitution burden across all normal liver tissues, blood, and tumors, as well as additional variants in tumors (Fig. 2B; Table S4). Compared to mutation burdens of corresponding untreated tissues, there was a several fold increase in substitution load in chemotherapy-treated tissues (9-fold for blood, 12-fold for normal liver, 5-fold for liver tumors), elevating the load of platinum-exposed tissues to that of adult liver (35) and blood (36) (Fig. 2B). Similarly, we observed increased burdens of indels and double base substitutions (DBS) in exposed normal tissues and tumors (Fig. S2A-C). There were no systematic regional differences in substitution load within the normal liver except for in one case (PD57226, Fig. S2D-E).

### Distinctive mutagenesis in the liver

Next, we examined mutational signatures of single base substitutions (SBS), as defined by the trinucleotide context of substitutions, using two orthogonal analytical methods, mSigHdp (37) and SigProfilerExtractor (38). Conventional bulk WGS of the tumor samples describes the history of the tumor (the most common ancestor cell the tumor bulk is derived from), but not its current per cell mutational state (in the absence of treatment-related clonal expansions) (Fig. 1A-B). Accordingly, in mutation catalogues derived by conventional WGS there were no treatment-related signatures in pre-treated tumor samples. Instead, they exhibited mutation signatures SBS1 and SBS5, which are ubiquitous and have been attributed to endogenous processes (7) (Fig. S3A-B). We could not extract signatures from normal blood and liver, due to the paucity of variants that conventional WGS delivered. In contrast, signature extraction of variants called by whole genome NanoSeq, which has single DNA molecule resolution and is independent of clones, revealed platinum agent mutagenesis (SBS31, SBS35) across pre-treated tissues, both normal and cancerous (Fig. 2C-E; Fig. S4). In exposed normal liver and hepatoblastoma tumors, but not in blood, both methods extracted a new mutational signature (SBSA; Fig. 2D-E; Table S5), meaning an imprint that could not be satisfactorily explained by the existing compendium of signatures (as per COSMIC, Fig. S5A-B) or by other platinum agent signatures documented in the literature (Fig. S5C). SBSA was even visible by eye in normalized trinucleotide spectra (mutational spectrum; Fig. 2C). It accounted for up to 92 % of single base substitutions (range 0-92 %; median 46 %) in each exposed normal liver sample (Fig. 2E). Like other mutagen-associated imprints, SBSA exhibited transcriptional strand bias (Fig. S5D). Its characteristic features were C>A (blue), C>G (black), and C>T (red) mutations in a GCC trinucleotide context (Fig. 2D; Fig. S6). The reliability of the extracted signature was high: 99 % by both mSigHdp and SigProfilerExtractor. The signature analysis was otherwise as expected, bar several biopsies from one tumor (PD62345), which exhibited a signature (SBS20) proposed to be caused by defective mismatch repair. Signatures of indels (Fig. S7) and DBS (Fig. S8) showed signatures consistent with previous studies (5) of pre-treated (including platinum agents) tissues. We did however see that the DBS mutational footprint differed between blood and liver (Fig. S8), similar to what we observed for SBS signatures.

### Validation in 255 tissue samples by targeted NanoSeq

To validate and extend our findings, we deployed targeted NanoSeq, which enabled us to assess both mutation burden and the potential functional impact of variants in genes of interest. We targeted a total of 285 genes (Table S6) comprising mostly cancer genes (39) as well as 7 genes reported to harbor somatic mutations in metabolic liver disease of adults (40). We first established whether targeted NanoSeq faithfully recapitulated findings from whole genome NanoSeq. We subjected the same stock DNA we had used for whole genome NanoSeq to targeted NanoSeq and found a high correlation of the mutation load (Pearson correlation = 0.95, p = 2.2 × 10^-16^, t-test; Fig. S9).

**Fig. 3:**
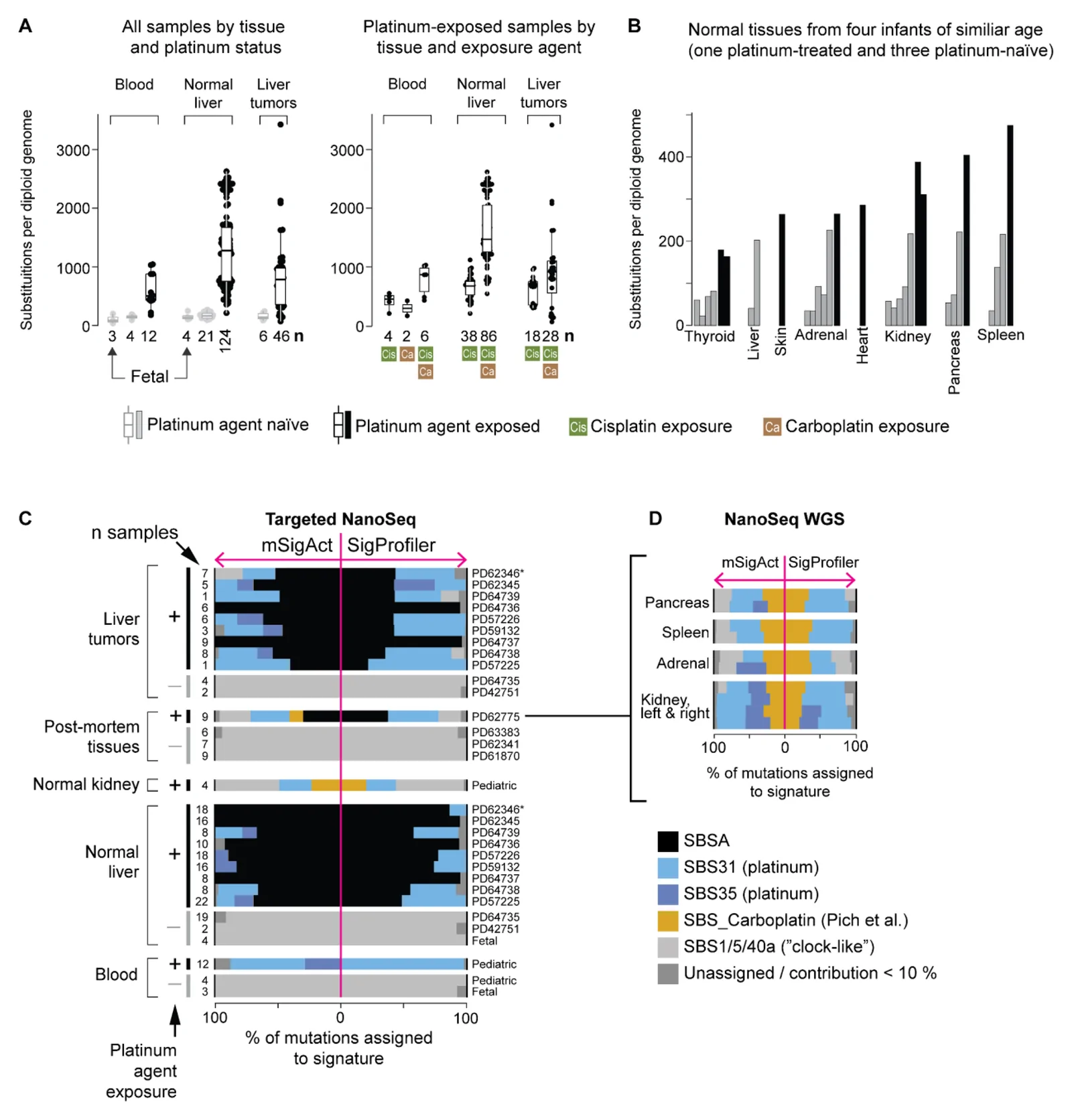
Mutation burden and signatures in validation cohort assessed by targeted NanoSeq. **(A)** Targeted NanoSeq of 220 samples (including 120 samples from the discovery cohort). X-axis: Samples grouped by tissue type (blood, normal liver and liver tumors) and platinum exposure, as labelled. n = number of samples per category. Y-axis: Number of single base substitutions per diploid genome. Platinum exposure is indicated by color (gray – platinum agent naïve; black – platinum agent exposed). Left: All 220 samples grouped by tissue type. Right: 181 samples grouped by platinum exposure. **(B)** Number of single base substitutions per diploid genome (Y-axis) in 31 different normal tissue samples obtained at post-mortems from four infants whereof one had received platinum-based treatment (PD62775). The infants were of a similar age. Exposed tissues were obtained from one infant who had been treated with one cycle of carboplatin. Exposure represented in colors (gray – platinum agent naïve; black – platinum agent exposed). **(C)** Contribution of signatures to each sample. X-axis: % of mutations assigned to signatures with each signature represented by a different color. Stacked bar plots to the left of the pink line are results from mSigAct whereas stacked bar plots to the right are results from SigProfilerAssignment. Y-axis: Each stacked bar plot represents combined tissue samples as per labelling. Note that liver tissue was not available from PD62775. * PD62346 is a recurrent hepatoblastoma. **(D)** Re-examination of normal tissues from PD62775 by whole genome NanoSeq (NanoSeq WGS). Axes as per (C). Signature analysis of a greater number of NanoSeq WGS mutations resolves conflicting results of targeted NanoSeq (average of 58 mutations per sample by targeted NanoSeq vs average of 398 mutations per sample by NanoSeq WGS) and shows that SBSA is absent from tissues from PD62775.

We then applied targeted NanoSeq to a total of 255 tissue samples (validation study, Fig. 1C) obtained from liver resections, pediatric post-mortems, and human fetuses, examining in total ∼115,000 diploid cells (255 tissues x median of 906 duplex coverage), and identified a total of 75,104 variants which we list in Table S7. Other than blood and liver, we studied the following normal tissues: spleen, pancreas, kidney, thyroid, skin, adrenal, and heart. We performed signature assignment of the targeted NanoSeq data using two orthogonal methods, SigProfilerAssignment (41) and mSigAct (42), testing for the presence of SBSA, previously described platinum signatures (SBS31, SBS35, SBS_Carboplatin (5)) and “clock-like” (3) signatures (SBS1, SBS5, SBS40a). Several patterns emerged from our analysis of mutation burden and corresponding signatures. First, platinum agent exposure increased the mutation load of all samples (Fig. 3A). Even short treatment durations increased the mutation load, as exemplified by an infant who received only a single cycle of carboplatin treatment (PD62775), which led to a four-fold increase in single base substitutions in normal tissues compared to three platinum-naïve infants of a similar age (Fig. 3B). Second, chemotherapy mutation load increased with exposure, as, for example, evident in children who received cisplatin alone in comparison to those who received cisplatin and carboplatin (Fig. 3A). Third, in this validation cohort we found SBSA again evident in platinum-exposed liver (Fig. 3C), but not in exposed blood or in other pediatric tissues (adrenal, kidney, pancreas, spleen, thyroid, heart, skin, platinum-naïve liver). These included tissues exposed to carboplatin alone (PD62775) where signature analysis initially yielded conflicting results, likely resulting from low mutation counts detected by targeted NanoSeq. We resolved these discrepant findings through whole genome NanoSeq to obtain a greater number of variants (Fig. 3D). Signature analysis in this larger number of genome-wide mutations revealed a previously described carboplatin-specific signature (SBS_Carboplatin (5); Fig. S10) with no evidence of SBSA in these tissues (note that no liver sample was available from this child). Further, SBSA was present in liver tissues from children who received cisplatin alone as well as in combination with carboplatin, indicating that cisplatin alone suffices to generate SBSA. We also detected SBSA in tissues of children who had not received the chelating agent sodium thiosulfate, which is usually co-administered with cisplatin to protect hearing (43), thus ruling out sodium thiosulfate as the cause of the new signature.

### Validation in external data sets, cell lines, and mice

To further validate our finding of a new mutational signature (SBSA), we examined external substitution catalogues derived from normal and cirrhotic adult liver (platinum-naïve), and from childhood cancers, including recurrent and metastatic childhood liver tumors (hepatoblastoma and hepatocellular carcinoma). Tumor recurrences and metastases may serve as reporters of past exposures, detectable by conventional bulk WGS, when they are derived from a single cell (rather than a group of cells) (Fig. 1A-B). To illustrate this point, we examined a recurrent hepatoblastoma in our cohort (PD62346) by conventional WGS, which demonstrated signatures of platinum agent exposure as well as SBSA in the tumor samples (Fig. S11A). Prior to the resection of the recurrent hepatoblastoma, this child had received further platinum chemotherapy which therefore generated additional variants, as revealed by whole genome NanoSeq (Fig. S11B).

**Fig. 4:**
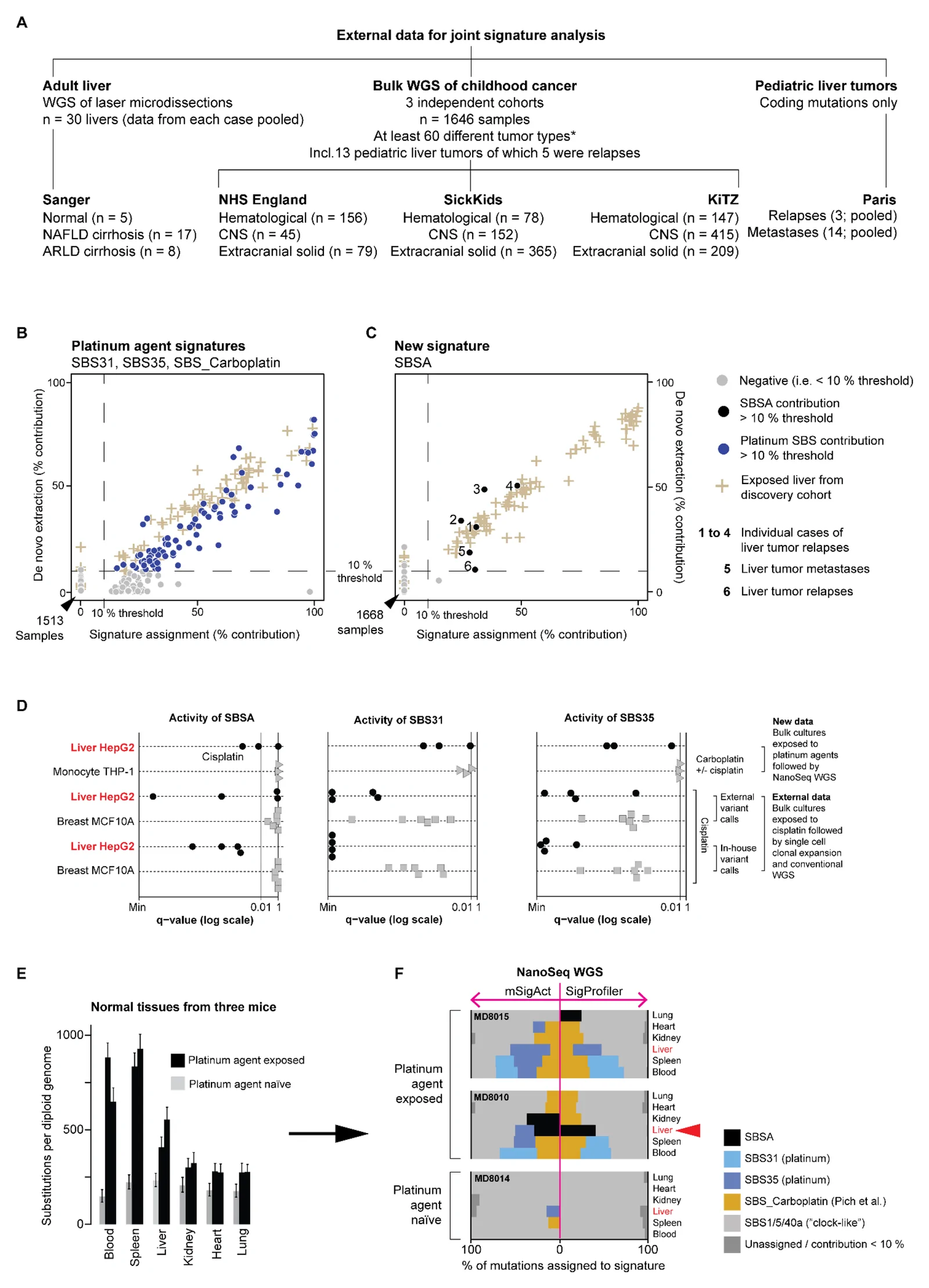
Signature extraction and assignment from mutation data of five relevant external cohorts, in vitro and in vivo experiments. **(A)** Description of different data sets. Note that the childhood cancer data contained five recurrent childhood liver tumors from children who would have been treated with platinum agents prior to relapse. **(B)** Contribution of established platinum agent signatures to each sample. X-axis: contribution as per signature assignment by SigProfilerAssignment (using signatures SBS1, SBS5, SBS40a, SBS31, SBS35, SBSA, SBS_Carboplatin (5)). Y-axis: contribution as per de novo extraction by mSigHdp. The dotted line in the plot represents the 10 % threshold generally considered the level above which a signature result is considered to be robust. Symbols and color coding as per legend. **(C)** Contribution of SBSA to each sample. Legends as per (C). Note that liver tumor metastases and liver tumor recurrences from the Paris cohort consisted of pooled data from 14 and 3 patients respectively. **(D)** Presence of SBSA investigated using a signature presence test (mSigAct package) testing the likelihood of reconstructing the mutational spectra with and without SBSA (left), SBS31 (middle) and SBS35 (right). The resulting p-value of the likelihood ratio test was adjusted to q-values using Benjamini & Hochberg. X-axis: log q-value for SBSA, SBS31 and SBS35 respectively. We generated whole genome NanoSeq (“New data”) from exposed cell lines, and included external conventional WGS data from single-cell-derived-clones of cisplatin-exposed breast (n = 6 clones) and liver (n = 4 clones) cell lines from Boot et al. (4), using both their external variant calls and variants reprocessed through our in-house pipeline. The line at 0.01 represents the threshold below which signatures are considered significantly present in a sample. The minimum q-value (Min) ranged from < 10^−16^ to < 10^-310^ and cannot be compared across experiments. Owing to the likelihood-based test, orders-of-magnitude differences arise that do not scale linearly with biological signal or imply proportional differences in signature strength. **(E)** Number of single base substitutions per diploid genome (Y-axis) in 31 normal tissue samples obtained from two mice exposed to cisplatin and carboplatin, and one platinum-naïve mouse. Exposure represented in colors (gray – platinum agent naïve; black – platinum agent exposed). **(F)** Contribution of signatures to each sample from two platinum-exposed (cisplatin and carboplatin) mice and one platinum-naïve mouse. Only one sample per tissue and mouse was analyzed; the in vivo experiment is intended as supportive evidence and not a quantitative assessment of penetrance. X-axis: % of mutations assigned to signatures with each signature represented by a different color. Stacked bar plots to the left of the pink line are results from mSigAct, whereas stacked bar plots to the right are results from SigProfilerAssignment. Y-axis: Each stacked bar plot represents a tissue sample as per labelling. Liver tissues are indicated with red text. Red arrow indicates the tissue where SBSA was confidently (by both methods) detected.

In total we studied mutation data from five external cohorts: normal and cirrhotic liver conventional WGS data (40); conventional WGS data from three different childhood cancer cohorts which included relapses of liver tumors (29, 44); and conventional whole exome sequencing data (45) which included relapses and metastases of childhood liver tumors (Fig. 4A). We assessed published variant calls for mutational signatures, deriving consensus calls from de novo extraction and signature assignment. Note that amongst 1,646 neoplasms, encompassing at least 60 different tumor types, there were 5 childhood liver tumor relapses that have been exposed to platinum agents during the treatment of the initial disease. De novo extraction and signature assignment singled out four of these (Fig. 4B-C) as harboring SBSA. Mutational signatures of the fifth liver tumor relapse (Fig. S12) were unusual and indicative of a signature reported for ganciclovir exposure (46). In addition, de novo extraction and signature assignment demonstrated SBSA in pooled coding mutations from childhood liver metastases and from recurrences (Fig. 4C). There was no evidence of SBSA in any other childhood cancer (including non-exposed liver tumors) or in adult cirrhotic liver samples based on conventional WGS/WES analyses (Fig. 4C).

To further validate the signature itself and its specificity for the liver, we pursued in vitro and in vivo experiments. First, we treated an adult liver cancer cell line and a monocyte line with platinum agents (cisplatin +/- carboplatin) followed by whole genome NanoSeq, which demonstrated the presence of SBSA in two liver cell lines treated with cisplatin alone and the combination of carboplatin and cisplatin, but not in exposed monocyte lines (Fig. 4D; Table S8). The liver cell line exposed to carboplatin only did not harbor SBSA. Next, we examined published data from an independent study (4) in which a liver cancer cell line and breast cell line had been treated by cisplatin, followed by single cell clonal expansion to reveal signatures (assessed by conventional WGS). We examined substitutions provided by the authors of the study (Table S9) and mutations that we called through our pipeline from reprocessed raw sequencing data (Table S10). We detected SBSA in both sets of substitution catalogues from cisplatin-treated liver cancer cell lines, but not from exposed breast cultures (Fig. 4D).

Next, we explored whether SBSA may also be present in murine tissues and studied blood, spleen, liver, kidney, heart, and lung derived from mice exposed to cisplatin and carboplatin (two exposed, one treatment-naïve). Normal tissues of exposed mice harbored elevated mutation burdens across all tissues compared to the platinum-naïve tissues (Fig. 4E, Fig. S13; median fold increase 1.8; range 1.5-5.2 fold increase per tissue; Table S11). We detected known platinum signatures (SBS31, SBS35, SBS_Carboplatin (5)) across all tissues. The only tissue that exhibited SBSA by two different methods was the liver of one mouse (Fig. 4F). This mirrors observations in platinum-exposed pediatric liver samples, where SBSA is not detected in every individual liver specimen (Fig. 2E).

Together these multiple orthogonal lines of enquiry validate SBSA itself, its association with cisplatin exposure, and predilection for liver-derived tissues by a variety of sequencing approaches, in human tissues, in cell lines, and in mice (see Supplementary Text detailing the various levels of validation).

### Generation of pathogenic mutations

**Fig. 5:**
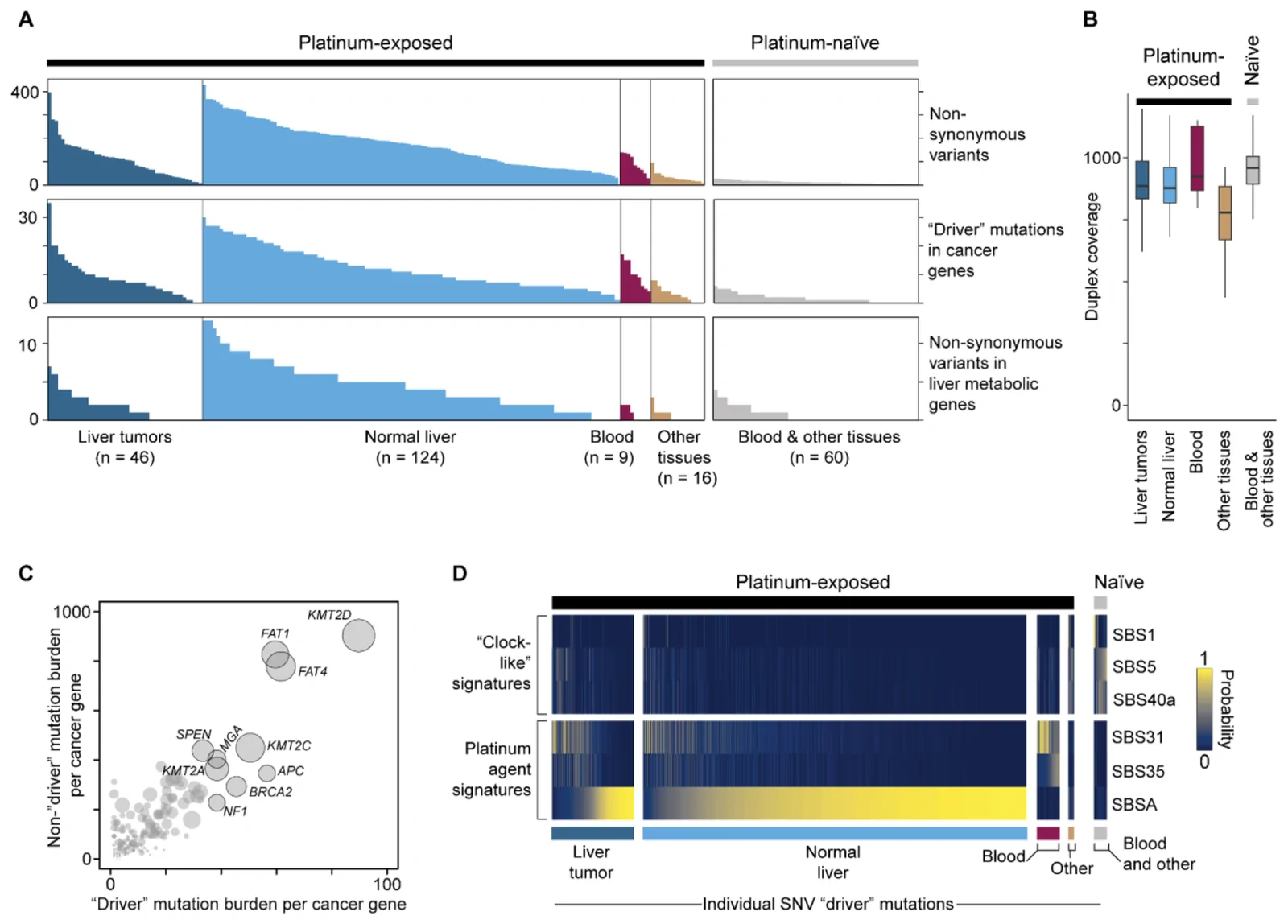
Annotation of the functional potential of variants called by targeted NanoSeq. **(A)** Number of variants (Y-axis) per sample (X-axis; each bar represents one sample) in descending order and grouped as per labels. Top: all non-synonymous variants. Middle: variants that would be considered driver mutations in the context of cancer. Bottom: non-synonymous variants in genes in which mutations have been causally linked to adult metabolic liver disease (40) (genes *ACVR2A, ALB, CIDEB, FOXO1, GPAM*, and *TNRC6B*). There was a significant increase in variants comparing exposed with non-exposed tissues in all three panels (p = 2.2 × 10^-16^; Welch two-sided t-test). Note that the order of the samples in the three panels is not the same. **(B)** Duplex coverage across groups. The duplex coverage is not higher in platinum-treated tissues than naïve tissues, and, therefore, does not cause the observed increase in non-synonymous variants and drivers in exposed tissues. **(C)** Correlation between non-driver mutation burden and potential driver mutation burden. Each circle represents a gene, and the size of the circle indicates the coding sequencing length of the gene. In total, there were 2,155 potential driver variants and 23,346 coding non-driver mutations in 194 cancer genes (gene list constrained to those with at least one potential driver variant). Pearson correlation = 0.85. Genes with a potential driver mutation burden above the 95th percentile are labelled by name. **(D)** Heatmap displaying the probability of potential cancer drivers (caused by a single base substitution; n = 1,228) to have arisen due to a specific mutational signature. The variants are categorized by tissue type and their platinum exposure status as displayed in A. The majority of potential cancer drivers in platinum-exposed tissues were generated by SBSA or the known platinum signatures (SBS31 and SBS35).

Targeted NanoSeq enabled us to examine whether chemotherapy mutagenesis generated variants with potential functional impact (e.g., non-synonymous mutations), which in some contexts may be considered pathogenic in mutation-driven disease. We interrogated a total of 285 genes (Table S6) including established cancer genes (n = 231) (39) and genes that have been found to harbor an excess of non-synonymous mutations in adult livers (n = 7), some of which have been implicated as contributing to metabolic liver disease (40). We divided coding variants (Table S12) into synonymous and non-synonymous. We then evaluated which non-synonymous variants in cancer genes may be consistent with variants considered pathogenic (“drivers”) in cancer (for example, truncating variants in tumor suppressor genes or hotspot mutations in oncogenes). It is important to note that a variant annotated as a driver in cancer may not necessarily have functional consequences in a normal cell (for example, an inactivating mutation in one allele of a tumor suppressor gene when the status of the second allele is unknown). Driver variants that we describe in normal tissues should, therefore, be interpreted as having potential, but not definitive, functional impact. In exposed compared to non-exposed tissues, we found a significant enrichment of non-synonymous variants in general (p = 2.2 × 10^-16^), of non-synonymous variants in genes related to metabolic liver disease (p = 2.2 × 10^-16^), and of cancer driver mutations (p = 2.2 × 10^-16^, Welch two-sided t-test for all three tests; Fig. 5A). Driver variants included tissue-relevant mutations, such as leukemogenic events in blood or in normal liver, and exon 3 hotspot mutation of *CTNNB1* (the principal driver of hepatoblastoma). The enrichment of driver variants in exposed tissue could not be explained by technical variation in sequence coverage (Fig. 5B). It likely derives from the increased mutation burden associated with platinum exposure, with larger genes being more vulnerable to variation than smaller ones (Fig. 5C). Consistent with this proposition, we found that SBSA accounted for the majority of cancer driver variants (Fig. 5D; Supplementary Text; Table S13). Furthermore, we did not find evidence that any particular cancer gene was under positive selection (Table S14), as assessed from the ratio of non-synonymous over synonymous variants using an established statistical framework (47). However, absence of selection should be interpreted in the context of the short interval (weeks) between the last exposure and surgery. While platinum exposure generates a large number of non-synonymous variants, the time frame is likely too short for the selective advantage of mutant clones to emerge.

Similarly, comparison of bulk transcriptomes from 86 platinum-treated and 16 untreated liver samples revealed statistically significant differences between groups (Fig. S14; Table S15). However, these were not readily attributable to platinum treatment and likely reflect underlying biological heterogeneity, including variability in tissue composition, rather than a treatment-specific transcriptional response. One non-cancer gene that exhibited a significantly increased ratio of non-synonymous variants in all liver tissues, whether exposed (corrected p-value = 3 × 10^-7^, likelihood-ratio test) or non-exposed (corrected p-value = 6 × 10^-5^, likelihood-ratio test), was the albumin gene, *ALB* (Fig. S15). This increase has previously been observed in adult liver cancer and in non-transformed adult liver tissue (48) and is of uncertain relevance.

## Discussion

The key finding of our study was that platinum chemotherapeutics, cisplatin and carboplatin, cause extensive and differential mutagenesis in normal tissues of children. Exposure to these agents mutationally “aged” pediatric tissues, generating variant burdens comparable to those seen in corresponding adult tissues, with differential mutagenesis in liver-derived tissues. Of note, targeted NanoSeq of cancer genes indicated the widespread presence of variants with features consistent with those considered pathogenic in the context of neoplasia, such as leukemogenic mutations. Overall, our findings reveal the vast extent of mutational damage that platinum agents may impart on normal tissues, which may plausibly underpin some of the late sequelae amongst survivors of childhood cancer.

The importance of our findings does not lie in the discovery of SBSA itself but in its predilection for liver-derived tissues, which we validated through multiple orthogonal approaches: multiple donors, different sequencing approaches, external data sets, in vitro experiments, and in mice. Tissue-specific mutagenesis has been observed with localized mutagens, such as UV light mutagenesis in skin (3, 49). It is, however, notable that a systematic mutagen penetrating all tissues in the body causes a tissue-specific mutational signature. In the context of the function of the liver, it would seem intuitive to implicate hepatic metabolism in the generation of SBSA, akin to liver mutagenesis caused by metabolism of aflatoxins (carcinogens associated with adult liver cancer) (50, 51). Platinum agents undergo breakdown in the liver, where a series of reactions produce a circulating metabolite that is primarily secreted via the kidney into urine (52, 53). We did not find SBSA in exposed blood or other organs, indicating that if a metabolite were the culprit, it would probably be an intermediary that is contained within the liver. SBSA arose in exposed cultures of liver cancer cells alone, indicating that liver cells on their own, without surrounding stroma, may generate SBSA. This does not exclude the possibility that factors other than hepatic metabolism may be contributing to SBSA in patients. SBSA was consistently associated with cisplatin exposure (including cisplatin alone) across experiments and data sets, whilst carboplatin alone may not suffice to generate SBSA, as seen in a liver cancer cell line treated with only carboplatin. Limitations of this study include the use of HepG2 cells, a cancer-derived liver cell line that may not fully represent normal hepatocyte function, and the small number of animals used for in vivo validation (n = 2 platinum-exposed and 1 platinum-naïve).

Deploying targeted duplex sequencing, we identified a link between chemotherapy mutagenesis and the generation of variants with potential functional impact, including mutations consistent with cancer “driver” mutations, in blood and liver. For example, in exposed normal liver amongst 108,872 molecules of DNA (124 samples x median of 878 duplex coverage), we found 21,714 non-synonymous variants and 1,522 potentially pathogenic variants residing in cancer genes. This would suggest that within the millions of cells of a whole human liver, chemotherapy is likely to have generated a vast repertoire of potentially pathogenic mutations. Some of the diseases that survivors of childhood cancer experience in later life, such as second cancers and some metabolic liver diseases (18, 19), are caused by somatic mutations. It is conceivable therefore that an increase of potentially pathogenic variants in normal tissues of exposed children elevates the risk of developing mutation-driven disease. If so, chemotherapy mutagenesis may represent a direct and quantifiable cause of late adverse effects from cancer treatment in childhood.

## Materials And Methods

### Ethics statement and sampling

Blood and tissue samples from children with liver tumors operated at the Institute of Liver Studies at King’s College Hospital were supplied via the King’s Paediatric Liver Research Biobank (REC ref: 23/WA/0059). Patients or guardians provided informed written consent for the storage and use of donated samples for research, with these samples subsequently released to this study as stipulated by the study protocol.

Fetal liver samples were obtained from the MRC–Wellcome Trust - funded Human Developmental Biology Resource (HDBR; http://www.hdbr.org) with appropriate written consent and approval from the North East – Newcastle & North Tyneside 1 Research Ethics Committee. HDBR is regulated by the UK Human Tissue Authority (HTA; www.hta.gov.uk) and operates in accordance with the relevant HTA Codes of Practice. We enriched these fetal samples for hepatocytes through sorting of magnetic beads into CD45+ and CD45- fractions prior to nucleic acid extraction and sequencing.

All other human tissues were collected through studies approved by UK NHS research ethics committees. Patients or guardians provided informed written consent for participation in this study as stipulated by the study protocols. These studies have the following references: NHS National Research Ethics Service reference 16/EE/0394; 16/LO/0960.

The in vivo experiments in mice were undertaken in compliance with UK Home Office legislation under the Animals (Scientific Procedures) Act 1986 under project license number PP3464389 and following the ARRIVE guidelines.

### Nucleic acid extraction

Samples were stored fresh-frozen in -80 °C until nucleic acid extraction according to standard methods using the Qiagen EZ1&EZ2 DNA Tissue kit for all human tissue samples, except for blood where the Qiagen EZ1&2 DNA Blood kit was used. The same stock of DNA was used for conventional WGS, whole genome NanoSeq and targeted NanoSeq.

### Conventional whole genome sequencing and filtering of variants

Short insert genomic libraries and flowcells were prepared followed by 150 bp paired-end sequencing clusters that were generated on Illumina HiSeq X. Resulting DNA sequences were aligned to the GRCh38 reference genome with the Burrows-Wheeler algorithm (54).

Variants were called using extensively validated pipelines at our Institute: substitutions were called by CaVEMan (34), indels by Pindel (55), and copy-number changes by ASCAT (56). CaVEMan and Pindel were run unmatched against an in silico human reference genome. Indel variants were filtered out if they failed Pindel’s filter or if they had a quality score below 300. Substitutions that passed CaVEMan’s filters were brought forward and mapping artefacts were removed by setting a threshold of ASMD ≥ 140, meaning the median alignment score of reads supporting a variant, and CLPM = 0, which requires that fewer than half of the reads were clipped. Germline substitutions and indels were filtered out by a one-sided exact binomial test as described previously (57). Substitutions in diploid regions with consistently high or low number of reads across normal samples from the same individual were removed. Next, all remaining substitutions and indels in samples from the same individual were recounted twice: once where we set a threshold of read mapping quality = 30 and base quality = 25 (high-quality reads), and once without applying any thresholds for mapping and base quality (total reads). Variants were included if the fraction of high-quality reads over total reads was greater than 75 %. To separate true substitutions from noise, we employed a beta-binomial model as described previously (57), where the locus-specific error rate is calculated using blood samples from healthy individuals as the reference. The resulting p-values were corrected for multiple testing with the Benjamini–Hochberg procedure and a cut-off was set at q < 0.01. Additionally, artefactual substitutions were removed if they were within 10 base pairs of where an indel had been called. Indels were removed if any indel had been called at the same position in the matched blood sample, and artefacts were also removed if any failed indel (indicative of a position with worse quality or mapping issues) had been called at that position in any other sample. Finally, all variants were manually inspected with jBrowse (58) to remove any artefacts remaining after filtering.

### Conventional whole genome sequencing - Variant calling of external cisplatin exposed cell lines

Fastq files were downloaded from the European Nucleotide Archive (project PRJEB21971; (4)) and aligned to the GRCh38 reference genome with the Burrows-Wheeler algorithm (54). Variants were called as described under “Conventional whole genome sequencing and filtering of variants”. Briefly, substitutions were called by CaVEMan, mapping artefacts were removed by setting a threshold of ASMD ≥ 140 and CLPM = 0, reads with consistently high or low depth were removed, recounting of reads with a read mapping quality > 30 and base quality > 25 (high-quality reads) and only keeping variants if the fraction of high-quality reads over total reads was greater than 75 %. Specifically for this dataset, we applied the following additional filters. Duplicated variants across clones from the same cell line were excluded. Variants with VAF < 0.2 were also removed (per the original paper), which is common practice for WGS data of single-cell derived clones (6, 12, 59–62) as these are considered as mutations acquired during in vitro expansion and/or technical artefacts.

### NanoSeq – library preparation, sequencing and variant calling

Whole genome NanoSeq libraries were prepared as previously described (32) and sequenced to 30 duplex coverage on Illumina NovaSeq 6000. Targeted NanoSeq libraries were prepared according to Lawson et al. (33), with 2 rounds of pulldown using a custom bait set (TWIST) consisting of 285 genes (∼ 1 MB in size; Table S6) and sequenced on Illumina NovaSeq 6000 (median duplex coverage ∼ 900 per sample). Sequenced DNA reads were aligned to GRCh37 (hs37d5 build) for targeted NanoSeq data and GRCh38 for whole genome NanoSeq using the Burrows-Wheeler algorithm. Variants (substitutions and indels) were called with the established NanoSeq pipeline as previously described (32; https://github.com/cancerit/NanoSeq). DBS were identified based on the passed substitutions using SigProfilerMatrixGenerator, which also created a DBS78 mutational matrix.

Conventional WGS of matched blood samples (median coverage of 88 x per sample) from the same individual was used as the matched normal in the variant-calling pipeline for whole genome NanoSeq, in accordance with published protocols (32, 33), to filter out germline variants. This approach removes only variants detected at high variant allele frequency (VAF > 10 %) in the matched normal (blood) by conventional WGS, which predominantly comprise germline variants and a small number of early post-zygotic mutations, while rare variants uniquely detected by whole genome NanoSeq remain unaffected. This is independent of platinum exposure, as conventional WGS lacks sensitivity to detect rare treatment-induced variants in the absence of clonal expansion. For targeted NanoSeq, high sequencing depth and captured polyclonality were leveraged to filter out variants with variant allele frequency > 10% (including germline variants), without the need for a matched normal sample, as previously described (33).

### NanoSeq – normalization of mutation burden and trinucleotide substitution profiles

Mutation rates are heavily affected by the trinucleotide context. We therefore normalized the obtained NanoSeq substitution burdens and trinucleotide frequencies to account for differences between the regions sequenced with NanoSeq and the genome-wide background, as described previously (32). The normalized trinucleotide frequencies and normalized substitution burdens are used throughout the paper. The normalized mutation burden is multiplied by the size of the human diploid genome (∼ 6 billion base pairs), or mouse genome where applicable (∼ 5.46 billion base pairs), to obtain the number of mutations per diploid genome, which is compared to the number of mutations obtained by conventional WGS.

### Filtering of targeted NanoSeq data

No samples for which data were generated were excluded from targeted NanoSeq analyses. Artefacts in targeted NanoSeq data may occur towards the start and end of reads, often because of germline indels at the edge of a read which can be miscalled as a substitution. To remove these, a Kolmogorov-Smirnov (KS) test on read position was applied across a large targeted NanoSeq dataset. Mutations were discarded if the KS test q-value was less than 0.05, and more than 50 % of the mutations were within the first or last 10 bp of callable reads. A subset of called mutations that had not been discarded were reviewed manually on jBrowse (58) and were consistent with true mutations.

### Driver annotation from targeted NanoSeq data based on prior knowledge

Variants were annotated with the dndscv package (47) and were annotated as “driver” mutations with functional potential if they fell into one of two categories: truncating mutations in tumor suppressor genes, or nonsynonymous mutations in known hotspots (positions within genes that are excessively mutated).

For the annotation of truncating mutations in tumor suppressor genes, four lists of tumor suppressor genes were defined: the COSMIC Cancer Gene Census (39), and lists based on curation of the literature for each of liver cancers, blood cancers, and Wilms tumors. The Cancer Gene Census should capture common pancancer genes, while tissue-specific lists were chosen based on the makeup of our dataset. A truncating (nonsense, frameshift, or splice site) mutation in any tumor suppressor in any of these lists was taken forwards as a candidate driver mutation with functional potential.

For the annotation of hotspot mutations, four lists of hotspot positions were generated using COSMIC data: across all cancer, and specifically in each of liver cancers, blood cancers, and Wilms tumors. Within each group, for every gene, the number of substitutions in every codon of the gene was counted. For each gene, the median number of mutations across all codons was calculated. If a codon had greater than four times the median plus one (one is added for cases where the median number of mutations is zero), or if it had more than 15 mutations in total, that codon was counted as a hotspot codon. Any nonsynonymous substitution in a hotspot codon was counted as a candidate driver mutation with functional potential.

### Selection analyses from targeted NanoSeq data

Selection analyses were carried out using the package dndscv (47) for the following nine sample groups: all samples together; normal liver with and without platinum exposure; all normal tissues with and without platinum exposure; blood with and without platinum exposure; and hepatoblastoma tumors with and without platinum exposure. For every group, dndscv was run with the following parameters, where the gene list included 280 protein coding genes included in the baitset:

dnds_output = dndscv(dndsin_input_mutations, max_muts_per_gene_per_sample = Inf, max_coding_muts_per_sample = Inf, gene_list = genelist, onesided = T, dc = duplex_coverage_vector, outmats = T).

Genes were considered to be under positive selection if their positive selection q-value across all mutation classes was < 0.05.

### Targeted NanoSeq – probability of a variant arising from a specific mutational signature

The probability of a certain substitution arising from a specific signature was calculated as previously described (27) where the weighted fraction of the signature contribution to the sample is multiplied by the mutational probability of the signature to cause the substitution in that specific trinucleotide context:

$$p_{C[C>T]C,sigA}=\frac{C_{sigA}\times P_{C[C>T]C,sigA}}{\sum C_{sigN}\times P_{C[C>T]C,sigN}}$$

I.e, the odds of signature A causing a C[C>T]C mutation is calculated by taking the contribution of signature A (C,sigA) to the sample (as defined by signature assignment or de novo extraction followed by decomposition) multiplied by the probability (P) that signature A generates a C[C>T]C change (percentage of SBS for a C[C>T]C change in signature A in COSMIC SBS96 reference data base). This is then normalized by the sum of these products for all signatures (N = number of signatures) detected in the sample of interest. As a result, the probability for each signature to have caused the substitution of interest will range between 0 and 1 with the highest score representing the most likely signature. An example of the calculation is provided in the Supplementary Text.

### De novo mutational signature extraction and decomposition

Mutations called as described above were assembled into SBS96, ID83 and DBS78 mutational matrices. De novo signature extraction for the three mutation types (SBS, indels and DBS) was then performed by two methods, which are both independent of the sequencing technology used to derive mutation calls. For both methods, signatures with a contribution lower than 10 % to a sample were considered unassigned. Method 1) SigProfilerExtractor (38) was run using default settings and a maximum of 20 signatures to be extracted. The suggested number of extracted signatures by the algorithm was used and decomposition into COSMIC reference version 3.4 was performed by the SigProfilerExtractor tool.

Method 2) mSigHdp (37) was run using the recommended values for gamma.beta (20 for SBS, 50 for indels and DBS extraction), number of burn-in (100,000 for SBS and DBS, 30,000 for indels) and number of chains/iterations (20 independent chains, 200 Gibbs samples per chain and with 100 iterations between collected samples). Three iterations of concentration parameters were performed after each iteration. The suggested number of extracted signatures by mSigHdp were used and decomposed into linear mixtures of COSMIC v3.4 reference signatures using an expectation maximization algorithm. Cosine similarity between the extracted signature and the reconstructed signature based on the decompositions were calculated both for the SigProfiler and mSigHdp extractions. Extracted signatures with a cosine similarity >= 0.9 were considered to be combinations of previously reported signatures, whereas a cosine similarity < 0.9 was considered insufficient to explain the extracted signature and thus suggestive of a novel extracted signature.

For the mSigHdp extraction of external datasets (sourced from the UK, Germany, Canada and France; Fig. 4A-C), we set downsampling to 3,000, as recommended to reduce computational demand for processing this highly mutated dataset and also reduced the number of burn-in to 10,000. SigProfilerExtractor (38) was used to extract signatures based on the SBS288 classification (an extension of the SBS96 classification that also considers the strand orientation of the mutation) to check for transcriptional strand bias of SBSA.

De novo signature extraction of the targeted NanoSeq data for the validation cohort was performed to assess SBSA, as extracted by targeted NanoSeq data, which was reassuringly very similar (cosine similarity 0.97) to SBSA as extracted from the WGS NanoSeq discovery cohort. The extraction was also performed to obtain SBS signature proportions that were used for subsequent calculations of driver probability (as described above). The validation cohort contains an overwhelmingly majority of platinum-exposed samples and mutations, and we therefore supplemented this cohort with 120 blood samples (Table S13) from platinum-naïve children with Wilms tumor to extract the clock-like signatures.

### Mutational signature assignment

We used two different methods for signature assignment of SBS1, SBS5, SBS40a, SBS31, SBS35 (all from COSMIC SBS96 reference version 3.4), SBSA as extracted by mSigHdp (from whole genome NanoSeq or targeted NanoSeq depending on the sequencing type of the samples), and SBS_Carboplatin (as extracted previously (5) using SignatureAnalyzer). The first method, SigProfilerAssignment (41), was run with the function cosmic_fit using default settings restricted to the aforementioned signatures. mSigAct (42) was applied using the function SparseAssignActivity, with its recommended settings and the p-value threshold was set to 0.05 divided by the number of signatures included for assignment. Both signature assignment methods are independent of if the mutations were called from conventional WGS or NanoSeq. Signatures with a contribution lower than 10 % to a sample were considered unassigned.

mSigAct (42) was also applied to the in vitro data set using the function SignaturePresenceTest, which tests whether a signature is plausibly present in a sample. It reconstructs the spectrum with and without the signature and performs a likelihood ratio test between the two scenarios which generates a p-value. The p-value was then corrected for multiple testing using the Benjamini and Hochberg method.

### In vitro cell line experiment

HepG2 (liver cell line) and THP-1 (monocyte-derived cell line) cell lines were obtained from the Francis Crick Institute Cell Science Scientific Technology Platform. HepG2 cells were maintained in DMEM with 10 % Foetal Bovine Serum (FBS), while THP-1 cells were cultured in RPMI 1640 supplemented with 10 % FBS at 37 °C with 5 % CO2 in a humidified cell culture incubator. Cisplatin (Cambridge bioscience, 13119-50mg) and carboplatin (Cambridge bioscience, 13112-25mg) were dissolved in Dimethyl Sulfoxide (DMSO), which was used as a vehicle control. 10^6^ HepG2 and THP-1 cells were seeded at day 0 in six-well plates. On day 1, cisplatin was added to a final concentration of 5 µM (for THP-1) or 10 µM (HepG2), carboplatin at 10 µM, cisplatin + carboplatin (10 µM each for HepG2, and 5 + 10 for THP-1). On day 2, the media containing the treatments was removed (24 hours treatment), cells were rinsed twice with Phosphate Buffered Saline and sufficient fresh media was added for maintenance of the cells for 6 days. On day 7, DNA was extracted from the treated cells with a Trizol (Fisher, 11588616) extraction. Whole genome NanoSeq was performed as described above and conventional WGS of the vehicle sample of the cell line was used as the matched normal in the variant-calling pipeline for whole genome NanoSeq to remove germline variants. Trinucleotide frequencies from vehicle samples were subtracted from those of treated samples, and any resulting negative values were set to zero prior to signature assignment.

### In vivo experiment in mice

We benefitted from an ongoing experiment at The Francis Crick Institute to collect unwanted tissues post platinum treatment from a xenograft study. NOD.Cg-PrkdcSCID Il2rgtm1Wjl/SzJ mice were bred at the Francis Crick Institute under specific pathogen-free conditions. Treatment and vehicle were randomized to remove any biases and all mice were handled blindly by qualified animal technicians throughout the xenograft study. 2 × 10^6^ cells of the SK-N-SH neuroblastoma cell line were injected subcutaneously with Matrigel (1 : 1 ratio) into the left mammary fat pad flank of 4-week-old female mice. 3 weeks post-injection (t0), mice were treated with platinum (cisplatin and carboplatin at 1.5 mg/kg and 30 mg/ml, respectively). Treatment was repeated 2 days later (t2) with only carboplatin, then on day 7 (t7) with only cisplatin, at t9 with carboplatin, at t14 with cisplatin and carboplatin, finally at t16 with carboplatin. A control mouse was injected with vehicle solution (saline) on the same days as the platinum treatments. Tumor sizes were measured 3 times a week and mice were euthanized once the mean tumor size reached 15 mm (vehicle control mouse on t12), or on termination point at t20 (2 x platinum-exposed mice). Liver, spleen, kidney, lung, heart and blood were harvested, and tissues were immediately snap-frozen in liquid nitrogen while blood was spun down to separate plasma from red blood cells. Tissues were then lysed using lysis buffer NTES (50 mM Tris ph 8, 50 mM EDTA, 100 mM NaCl, 1 % SDS and + 10 µl proteinase K and DNA was extracted through phenol-chloroform isolation method, precipitated with isopropanol and resuspended in water.

Whole genome NanoSeq was performed as described above and conventional WGS of the blood sample from each mouse was used as the matched normal in the variant-calling pipeline for whole genome NanoSeq to remove germline variants. In addition, the following filters were applied to the mouse NanoSeq data to remove noise and artefacts: 1) only unique calls per mice were retained, 2) calls which had any supporting reads in the WGS matched normal were removed, 3) calls with depth < 20 in the WGS matched normal were removed, 3) calls from reads containing > 1 mutation were removed. The mutation burden and trinucleotide frequencies were corrected as described above using the GRCm38 reference genome.

### Bulk RNA analysis

Bulk RNA libraries were prepared and sequenced on Illumina HiSeq 4000 platform. Sequencing reads were aligned with STAR (63) and mapped to GRCh38. HTSeq (64) was used to obtain read counts of genes. Data was processed using the R package EdgeR (65), normalized using the TMM method, and converted to log-CPM values. Limma (66) and Glimma (67) were thereafter used for determining differential gene expression (requiring a log fold change significantly > 1) between platinum exposed liver samples and platinum naïve liver samples. Genes with adjusted p-value < 0.05 were considered differentially expressed between the groups.

## Supplementary Material

Fig. S1

MDAR Reproducibility Checklist

Table S1

## Data Availability

De-identified patient-level information and variant calls are provided in Supplementary Tables. Sequencing data are available through the European Nucleotide Archive (ENA) and the European Genome-Phenome Archive (EGA) under the following studies/accession codes: EGAD00001016117 (RNA), EGAD00001016120 (targeted NanoSeq), EGAD00001016122 (WGS, patient material), EGAD00001016141 (WGS, cell lines), EGAD00001016142 (whole genome NanoSeq, patient material), EGAD00001016143 (whole genome NanoSeq, cell lines), study ERP183275 (mouse WGS), ERP181225 (mouse whole genome NanoSeq) and ERP192212 (targeted NanoSeq, fetal samples). External datasets analyzed in this study are either published (4, 29, 40, 44, 45), available upon request from Adam Shlien (SickKids WGS study) or provided as Supplementary Material (substitution calls from (4) kindly provided by Steve Rozen). Custom code is available at https://github.com/WengerAnna/platinum_liver and archived on Zenodo (68; https://doi.org/10.5281/zenodo.20374075). Cell lines and mouse strains used in this study are available through Foad Rouhani upon reasonable request, subject to institutional material transfer agreements.
