# Supplementary material for: Extensive and differential platinum chemotherapy mutagenesis in livers of children
Source: Science. Author manuscript; Available in PMC 2026 Nov 16. (PMC7619738; doi:10.1126/science.ady0339)
Supplement: Supplementary file 2 [file EMS222181-supplement-Fig__S1.docx]

Supplementary Materials for

**Extensive and differential platinum chemotherapy mutagenesis in livers of children**

Anna Wenger, Jean-Baptiste Vannier, Henry Lee-Six, Robin Loesch, Giulia Emanuelli, Manas Dave, Mehdi Layeghifard, Andrew R.J. Lawson, Federico Abascal, Pantelis A. Nicola, Taryn D. Treger, Toochi Ogbonnah, Conor Parks, Thomas R.W. Oliver, Jonathan Kennedy, Angus Hodder, Nathaniel D. Anderson, Felipe Luz Torres Silva, Mi K. Trinh, Thomas Dowe, Marwo Habarwaa, James J. Sun, Sergio Assia-Zamora, Miriam Cortes-Cerisuelo, Wayel Jassem, Charlotte Town, Anil Dhawan, Vandana Jain, Karin Straathof, Maesha Deheragoda, Iñigo Martincorena, Liina Palm, J. Ciaran Hutchinson, Tim H.H. Coorens, Claire Trayers, Mariia Yuneva, Nigel Heaton, Adam Shlien, Yoh Zen, Foad J. Rouhani^*^, Sam Behjati^*^

Corresponding authors: foad.rouhani@crick.ac.uk, sb31@sanger.ac.uk

**The PDF file includes:**

Supplementary Text

Figs. S1 to S15

References 69-79

**Other Supplementary Materials for this manuscript include the following:**

Tables S1 to S15

**Supplementary Text**

Limitations of conventional WGS

Conventional WGS has revolutionised our understanding of tumours and drivers of cancers (*39, 69*). However, owing to the fundamental limitations of WGS, as discussed in this note, the study of rare mutations or mutations present in single cells or small clones have been notoriously difficult. Determining mutation rates in polyclonal samples, such as normal tissues, has therefore required specialized approaches, including laser capture microdissection (*6*, *35*, *70*), or expansion of single cells in vitro (e.g.organoids or colonies) prior to sequencing (*62*, *71*).

A key challenge lies in accurately distinguishing a subclonal mutation of low variant allele frequency (VAF) from errors generated during library preparation and sequencing. Errors arising during PCR amplification in one DNA strand can be propagated and misinterpreted as a mutation by conventional WGS. Duplex sequencing approaches (such as NanoSeq (*32*, *33*)) overcome this by independently sequencing both strands of the same DNA molecule, enabling identification and removal of such artefacts (*32*, *72*). The exception is early and complementary PCR errors affecting both strands, although these occur at extremely low frequencies ( < 10^-9^ errors per base pair) (*73*).

The intrinsic error rate of conventional WGS sequencing, which is between 1 per 100 and 1 per 1,000 bases sequenced (*73, 74*), limits the detection of low-frequency variants. Mutations in tissues with a low mutation burden are therefore obscured among errors. As a comparison, the error rate for NanoSeq is < 5 errors per billion base pairs (*32*).

Various bioinformatic approaches and filters have been developed to minimise erroneous mutation calls and to try to flag artefacts. Some examples include:

1. Removing reads of lower quality (*75*).
2. Avoiding variants called in the beginning or end of reads (representing erroneous end-repair and mapping errors).
3. Requiring that the variant is called in independent reads, and in both directions (*76*).
4. Setting a base and mapping quality threshold (*34*).
5. Shearwater approaches (*57*, *77*) for testing if a variant in a sample is above sequencing error noise on that specific position in a reference panel of normal samples.

Bioinformatic filtering can substantially reduce artefacts but cannot fully overcome intrinsic error rates of conventional WGS (PCR amplification, sequencing errors etcetera). It still cannot accurately, and consistently, detect rare variants in polyclonal tissues, which is why duplex sequencing approaches are increasingly used for high-resolution studies of mutagenesis in normal tissues (*12*, *32*, *33*, *78*, *79*).

Validation of SBSA

Multiple lines of analyses and validation were pursued to corroborate the finding of SBSA. Here, we detail the full extent of these validation efforts and the potential causes that were systematically ruled out.

All tissues were handled in the same way and snap frozen in liquid nitrogen without any treatment. Tissues were collected from several different hospitals and pathology departments and we also validated SBSA in external datasets. It therefore seems highly unlikely that SBSA is caused by artefacts induced by tissue handling. Further, if tissue handling had caused artefacts we would have seen SBSA across all samples, not only in platinum-exposed liver samples.

Sequencing artefacts were unlikely to explain SBSA, as they would be expected to occur across all samples rather than being restricted to platinum-exposed liver. However, to formally rule out sequencing artefacts we used three different sequencing methods (and mutation calling pipelines): conventional WGS, whole genome NanoSeq (*32*) and targeted NanoSeq (*33*). The detection of SBSA across multiple sequencing methods, including external conventional WGS datasets analysed with independent pipelines, strongly argues against sequencing artefacts or mutation-calling approaches as the underlying cause. Conventional WGS only detected SBSA in recurrences as this technology requires a clonal outgrowth for reliable mutation calling (*31*).

Our finding of SBSA was further substantiated by the fact that it was extracted using two independent methods, which are based on different approaches:

1. SigProfilerExtractor is based on non-negative matrix factorization (NMF) (*2*, *38*).
2. mSigHdp relies on hierarchical Dirichlet process (HDP) mixture models (*37*).

Both methods extracted SBSA with a high confidence score (99 %), and the signature, as extracted by each method, was very similar (cosine similarity of 0.984). In addition to these two signature extraction methods, we also received concordant results upon utilising two signature assignment methods:

1. SigProfilerAssignment (*41*) that uses a forward stagewise algorithm for sparse regression and nonnegative least squares to optimize the signature assignment.
2. mSigAct (*42*) provides functionality for a maximum likelihood approach and conservatively estimating the minimal number of signatures needed to faithfully reconstruct the mutational spectra of a sample.

We included samples of different tissues, and with versus without platinum exposure (of different platinum agents), as well as in vitro and in vivo studies to pinpoint the cause of SBSA. We only found the signature in platinum-exposed liver samples and it was present for children who had received cisplatin alone and for children who had received cisplatin in combination with carboplatin. Some, but not all, children had also received sodium thiosulfate (STS), but it was ruled out as the cause as SBSA was present also in the children who did not receive STS. Taken together, this suggests that cisplatin alone may be enough to give rise to SBSA. This notion was further substantiated by the presence of SBSA in liver cancer cell lines (HepG2) treated with cisplatin, or the combination of cisplatin & carboplatin, but absence from carboplatin-exposed cells. SBSA was absent from all non-liver cell lines (breast and monocytes) for all tested platinum agents (including cisplatin). SBSA was also detected in the liver of one of two mice exposed with both cisplatin & carboplatin.

We excluded the possibility of SBSA being present in the liver in general (prior to platinum exposure) by examining fetal liver and platinum naïve liver tissue from children and adults (*42*) where SBSA was absent.

Normal tissue samples were obtained at post mortem from four children with non-liver cancer disease and included in the study. One of these children had received platinum-based therapy (carboplatin), but normal liver tissue was not available for analysis. However, we could not detect SBSA in any of the examined platinum-exposed normal tissues (adrenal, kidney, pancreas, spleen, thyroid, heart, skin) from this child, which was expected as the child had received carboplatin and no cisplatin. This, together with the fact that SBSA was absent from platinum (cisplatin or cisplatin & carboplatin) exposed blood, substantiates the notion of a signature with a predilection for liver-derived tissues. Further, we investigated external datasets (WGS or WES) of 1,646 neoplasms in children (*29*, *40*, *44,* *45*), encompassing at least 60 different tumor types, and only detected SBSA in recurrences of liver tumors and liver metastases.

Collectively, the various validation approaches described above indicate that SBSA is not artefactual but rather a bona fide signature of cisplatin exposure in liver tissue, with apparent tissue specificity across the platinum-exposed samples analyzed.

Example calculation: probability of variant arising from a specific mutational signature

The probability calculation is performed as described previously (*27*) and is based on the following formula:

$p_{G\left[ C>A \right]C, SBSA=\frac{C_{SBSA}\times P_{G\left[ C>A \right]C,SBSA}}{\sum C_{sigN}{\times P}_{G\left[ C>A \right]C,sigN}}}$

I.e, the odds of SBSA causing a G[C>A]C mutation is calculated by taking the contribution of SBSA (C,SBSA) to the sample multiplied by the probability (P) that SBSA generates a G[C>A]C change (percentage of SBS for a G[C>A]C change in SBSA in reference mutational matrix). This is then normalized by the sum of these products for all signatures (N = number of signatures) detected in the sample of interest.

For example, see below for the calculations of the probabilities for a TP53 driver substitution in the platinum-exposed normal liver sample PD57226b. The mutation is a C>A at GCC, with 96 % probability of being caused by SBSA.

| **Signature** | **Contribution to sample** | **Probability of G[C>A]C** | **Normalized probability** |
| --- | --- | --- | --- |
| SBSA | 0.776 | 0.776 * 0.180 = 0.1398 | 0.9556 |
| SBS35 | 0.066 | 0.066 * 0.050 = 0.0033 | 0.0225 |
| SBS31 | 0.143 | 0.143 * 0.021 = 0.0031 | 0.0211 |
| SBS40a | 0.005 | 0.005 * 0.011 = 5e-05 | 0.0004 |
| SBS5 | 0.008 | 0.008 * 0.008 = 6e-05 | 0.0004 |
| SBS1 | 0.002 | 0.002 * 0.0003 = 7e-07 | 5e-06 |

**Fig. S1. Driver variants and copy-number alterations in hepatoblastoma tumors.**

**(A)** Drivers (based on conventional WGS) in the tumors in the discovery cohort include 11p loss of imprinting (LOI) and loss of heterozygosity (LOH), as well as somatic substitutions and small insertions/deletions (indels).

**(B)** ASCAT copy-number plots (based on conventional WGS) of one representative tumor sample per child in the discovery cohort. A normal diploid genome without any copy-number aberrations has one copy each for every somatic chromosome (green and red line at 1). The tumors have common alterations in hepatoblastoma such as 11p LOH, gain of 1q, gain of 2q and gain of chromosome 20. * Mosaic 11p LOH is present in the germline and prevents 11p LOH from being detected in the tumor sample since the copy-number calling is run against the matched blood sample.

**Fig. S2. Indel and double base substitution burden in the discovery cohort.**

**(A)** Indel burden by conventional whole genome sequencing (WGS). X-axis: different tissues divided based on platinum exposure as shown in legend in (B). Y-axis: number of indels per diploid genome.

**(B)** Indel burden by WGS NanoSeq. Axes and horizontal lines as per (A).

**(C)** Double base substitution (DBS) burden by whole genome NanoSeq. X-axis: different tissues divided based on platinum exposure as shown in legend in (B). Y-axis: number of DBS per diploid genome.

**(D)** Single base substitution burden by whole genome NanoSeq for PD57226 shows regional variation in normal liver samples colored according to their sampling location in (E). Burdens are displayed only for normal liver samples.

**(E)** Sampling schematic of PD57226. Normal liver (N) and tumor (T) tissue. Yellow arrows delineate the tumor.

**Fig. S3. De novo signature extraction of single base substitutions called by conventional WGS.**

**(A)** mSigHdp de novo extraction and subsequent decomposition into known COSMIC SBS96 signatures of single base substitutions from liver tumors in the discovery cohort based on conventional WGS. Contribution of signatures to each sample. X-axis: Each stacked bar plot represents a tissue sample as per labelling. Y-axis: % of mutations assigned to signature. Pink square brackets delineate individuals. Colors represent mutational signatures as per legend.

**(B)** SigProfiler de novo extraction and subsequent decomposition into known COSMIC SBS96 signatures of substitutions from liver tumors based on conventional WGS. Same setup of plot as in (A). Note that SBSA was not detected by conventional WGS and neither were any of the known platinum signatures (SBS31 and SBS35).

**Fig. S4. Mutational spectrum of all samples in the discovery cohort called by whole genome NanoSeq.**

Plots showing the trinucleotide context (the reference base before and after a variant) of each single base substitution (X-axis) and the frequency of each trinucleotide (Y-axis) for each sample in the discovery cohort by whole genome NanoSeq.

**Fig. S5. De novo signature extraction of single base substitutions called by whole genome NanoSeq.**

**(A)** The five extracted de novo SBS96 signatures by mSigHdp (second column) based on whole genome NanoSeq data of the discovery cohort, and the known SBS signatures they decompose into (first column; the reconstructed cosine similarity is stated on top of the stacked bar plot). Decompositions with a reconstructed cosine similarity below 0.9 (marked in red) were considered as insufficient to explain the extracted signature. Colors of SBS signatures according to legend. The third column (gray box) shows the cosine similarity between the de novo signatures extracted by mSigHdp and SigProfiler (fourth column). The decomposition of the SigProfiler extracted signatures are shown to the far right (fifth column). The signatures extracted by the two methods were similar.

**(B)** Contribution of signatures to each sample. X-axis: Each stacked bar plot represents a tissue sample as per labelling. Y-axis: % of mutations assigned to signature. Pink square brackets delineate individuals. Colors represent mutational signatures as per legend. The SigProfiler extraction is shown here. Similar results were obtained by mSigHdp (Fig. 2E). Note that SBSA was only evident in platinum-exposed livers whereas conventional platinum signatures (SBS31 and SBS35) were pervasive across all platinum-exposed tissues.

**(C)** We included platinum signatures as described in the literature (*5*), but SBSA could not be decomposed into those and they do not explain SBSA.

**(D)** SBSA displays transcriptional strand bias.

**Fig. S6. Differences between SBSA and established platinum signatures in COSMIC.**

**(A)** Plot showing the trinucleotide context (the reference base before and after a variant) of each single base substitution (X-axis) and the frequency of each trinucleotide (Y-axis) for SBSA minus SBS31.

(**B**) Plot showing the mutational spectrum for SBSA minus SBS35.

**Fig. S7. De novo signature extraction of insertion/deletions called by whole genome NanoSeq.**

**(A)** The five extracted de novo indel signatures by mSigHdp (second column) based on whole genome NanoSeq data of the discovery cohort and the known ID83 signatures they decompose into (first column; the reconstructed cosine similarity is stated on top of the stacked bar plot). Decompositions with a reconstructed cosine similarity below 0.9 (marked in orange) were considered as insufficient to explain the extracted signature. Colors of ID signatures according to legend. The third column (gray box) shows the cosine similarity between the de novo signatures extracted by mSigHdp and SigProfiler (fourth column). The decomposition of the SigProfiler extracted signatures are shown to the far right (fifth column).

**(B)** Contribution of signatures to each sample. X-axis: Each stacked bar plot represents a tissue sample as per labelling. Y-axis: % of mutations assigned to signature. Green square brackets delineate individuals. Colors represent mutational signatures as per legend. The mSigHdp extraction is shown here.

**(C)** Similar results were obtained by the SigProfiler extraction.

**Fig. S8. De novo signature extraction of double base substitutions called by whole genome NanoSeq.**

**(A)** The two extracted de novo double base substitution (DBS) signatures by mSigHdp and SigProfiler based on whole genome NanoSeq data of the discovery cohort. One of the extracted signatures is extremely similar across the two methods (cosine similarity 0.99) whereas the other signature differs in terms of CC>NN mutations, but is otherwise similar between the two methods and thus visualised in the same color in (B) and (C). The extracted signatures are best explained by using DBS2 (included in the COSMIC reference database) and two platinum signatures extracted by Pich et al. (*5*) (right; indicated by *). The platinum signatures extracted by Pich et al. are a refinement of the platinum signature DBS5 present in the COSMIC reference database. The reconstructed cosine similarity of the decomposition of the mSigHdp extracted signatures is stated on top of the stacked bar plot. Colors of DBS signatures according to legend.

**(B)** Contribution of signatures to each sample. X-axis: Each stacked bar plot represents a tissue sample as per labelling. Y-axis: % of mutations assigned to signature. Pink square brackets delineate individuals. Colors represent mutational signatures as per legend. Samples with fewer than 10 DBS were excluded from (B) and (C). The mSigHdp extraction is shown here. Note that signature HDP N1 / DBS78A was only present in platinum-exposed normal liver and tumors, but largely absent from platinum-exposed blood. ** Samples with < 10 DBS were excluded from the plot.

**(C)** Similar results were obtained by the SigProfiler extraction. ** Samples with < 10 DBS were excluded from the plot.

**Fig. S9. Single base substitution burden in targeted versus whole genome NanoSeq.**

Mutation burden by targeted NanoSeq (Y-axis) versus mutation burden by whole genome NanoSeq (X-axis) shows a high correlation (Pearson correlation = 0.95, p = 2.2 x 10^-16^, parametric t-test). Platinum-exposed samples are colored in black and platinum-naïve in gray.

**Fig. S10. Trinucleotide context of carboplatin signature previously described in the literature.**

Plot showing the trinucleotide context (the reference base before and after a variant) of each single base substitution (X-axis) and the frequency of each trinucleotide (Y-axis) of the previously described carboplatin signature by Pich et al. (*5*).

**Fig. S11. Signature assignment and mutation burden in a recurrent hepatoblastoma.**

**(A)** Contribution of signatures to each sample based on conventional whole genome sequencing (WGS) of the recurrent liver tumor PD62346. X-axis: % of mutations assigned to signatures with each signature represented by a different color. Stacked bar plots to the left of the pink line are results from mSigAct whereas stacked bar plots to the right are results from SigProfilerAssignment. Y-axis: Each stacked bar plot represents a tissue sample as per labelling.

**(B)** Mutation burden by whole genome NanoSeq for platinum-exposed normal liver and tumors respectively divided into all primary platinum-exposed children (black) vs all platinum-exposed recurrent hepatoblastoma samples (PD62346; blue). Samples from the recurrence have significantly higher mutation burden than samples from primary cases. The brown horizontal line represents mutation burden observed in normal adult liver (*34*).

* p = 1.7 x 10^-5^, ** p = 2.2 x 10^-16^ (Welch’s t-test).

**Fig. S12. Mutational spectra and signature assignment of a recurrent hepatocellular carcinoma.**

**(A)** Trinucleotide spectra for zcc57 (top), a platinum-exposed recurrent hepatocellular carcinoma tumor, which showed no signal of SBSA. It displayed a spectrum similar (cosine similarity 0.98) to a signature attributed to exposure to ganciclovir (an antiviral medication) described by Kanter et al. (*46*; bottom).

**(B)** Signature assignment by mSigAct (left of the pink vertical line) and SigProfilerAssignment (right of the pink vertical line) both show that the ganciclovir signature is present in the zcc57 tumor sample. The cosine similarity from the assignments compared to the original spectra of the tumor was very high (0.99 for both), indicating an accurate assignment.

**Fig. S13. Trinucleotide spectra of normal tissues from two mice exposed to cisplatin and carboplatin.**

**(A)** Plots showing the normalized trinucleotide context (the reference base before and after a variant) of each single base substitution (X-axis) and the frequency (Y-axis) of each trinucleotide for platinum-exposed tissues (one tissue per row) from two mice (MD8010, left column; MD8015, right column).

**(B)** Hierarchical clustering based on cosine similarity of the two platinum-exposed mice. Color scale according to legend.

**Fig. S14. Differential gene expression analysis.**

Heatmap of differentially expressed genes (adjusted p-value < 0.05; n = 2,261 genes; Table S15) between 86 platinum-exposed liver samples (normal and tumor) and 16 platinum-naïve samples (normal and tumor) from the discovery cohort.

**Fig. S15. dNdS ratio of albumin gene.**

**(A)** dN/dS ratios for missense and truncating mutations across the whole genome and over just the gene encoding albumin (*ALB*) in liver and non-liver samples with and without platinum exposure. The central point represents the maximum likelihood estimate (MLE), and the bars represent the 95 % confidence interval.

**(B)** The proportion of mutations in each sample group that are missense, nonsense, synonymous, truncating, or splice site mutations. The sample group is shown below, with the number of samples included in each group, the total duplex coverage (the mean per-gene coverage summed across all samples within the sample group, summed across all genes in the ‘all genes’ analysis and just for *ALB* in the albumin-only analysis), and the total number of mutations that could be assessed by the dndscv algorithm.

Supplementary table titles and legends

**Table S1. Sample and sequencing information.**

**Table S2. Single base substitutions detected in blood, normal liver and liver tumors in the discovery cohort by conventional WGS.**

**Table S3. Driver mutations in liver tumors in the discovery cohort detected by conventional WGS**.

**Table S4. Unique single base substitutions and indels per sample detected in blood, normal liver and liver tumors in the discovery cohort by whole genome NanoSeq.**

**Table S5. Trinucleotide matrix for SBSA.**

**Table S6. Genes included in the baitset for targeted NanoSeq.**

**Table S7. Unique mutations per sample detected in the validation cohort by targeted NanoSeq.**

**Table S8. Trinucleotide matrix for platinum-exposed cell lines (whole genome NanoSeq) following subtraction of the untreated vehicle control.**

**Table S9. External substitution calls of WGS data from cisplatin-exposed cell lines.**

External substitution calls from single-cell-expanded cisplatin-treated clones of cell lines from Boot et al. (*4*). Variants are reported in hg37 coordinates.

**Table S10. In-house substitution calls of WGS data from cisplatin-exposed cell lines**.

In-house substitution calls from single-cell-expanded cisplatin-treated clones of cell lines from Boot et al. (*4*). Variants are reported in hg38 coordinates.

**Table S11. Trinucleotide matrix for mouse tissues.**

Two mice were platinum-exposed (MD8010 and MD8015) and one was platinum-naïve (MD8014). Samples were sequenced with whole genome NanoSeq. Trinucleotide frequencies were corrected based on the mouse genome, and variants were filtered.

**Table S12. Coding mutations detected in the validation cohort by targeted NanoSeq, annotated by dnds and analysed for functional potential.**

**Table S13. Platinum-naïve samples included with validation cohort for de novo signature extraction from targeted NanoSeq data.**

The obtained signatures were subsequently decomposed into COSMIC reference signatures and SBSA. The assigned contribution of each signature was used to calculate the probability that a variant was caused by the signature.

**Table S14. Output from selection analyses of targeted NanoSeq data using dnds.**

**Table S15. Differentially expressed genes between platinum-exposed and platinum naïve liver.**
